# Editorial: COVID-19 and the Digestive System

**DOI:** 10.3389/fmed.2022.875063

**Published:** 2022-03-30

**Authors:** Chunxiang Ma, Weiguo Dong, Bo Shen, Hu Zhang

**Affiliations:** ^1^Department of Gastroenterology, West China Hospital, Sichuan University, Chengdu, China; ^2^Centre for Inflammatory Bowel Disease, West China Hospital, Sichuan University, Chengdu, China; ^3^Lab of Inflammatory Bowel Disease, Frontiers Science Center for Disease-Related Molecular Network, West China Hospital, Sichuan University, Chengdu, China; ^4^Department of Gastroenterology, Renmin Hospital of Wuhan University, Wuhan, China; ^5^Inflammatory Bowel Disease Center, Division of Colorectal Surgery, Columbia University Medical Center/New York Presbyterian Hospital, New York, NY, United States

**Keywords:** COVID-19, SARS-CoV-2, endoscopy, liver, pancreas, gastrointestinal tract

The COVID-19 pandemic, caused by severe acute respiratory syndrome coronavirus 2 (SARS-CoV-2), remains persistent worldwide. Gastrointestinal (GI) symptoms, such as nausea, vomiting, diarrhea, and abdominal pain, have been frequently reported in COVID-19 patients. Angiotensin-converting enzyme 2 (ACE2), the functional receptor of SARS-CoV-2, has also been detected in the digestive system, indicating that this system is an infection route of COVID-19 besides the respiratory system (1, 2). In this Research Topic, specialists probe the involvement of the digestive system in COVID-19 from mechanisms to clinical practice.

Perisetti, Goyal, Gajendran, et al. present the rates of various GI manifestations in COVID-19 patients. The study by Chen R et al., with 1,133 hospitalized COVID-19 patients, further shows that severe cases are more frequently accompanied by GI symptoms. Compared to those without GI symptoms, COVID-19 patients with GI symptoms were not only more likely to develop adult respiratory distress syndrome (ARDS) and required non-invasive mechanical ventilation (Chen R et al.), but also had significantly prolonged hospital stays and higher hospitalization costs (Zhang et al.). However, the correlation between GI symptoms and the progression of COVID-19 is still controversial (3), probably because of the difference in research methods, sample sizes, and epidemic prevention policies between regions among studies. Moreover, medications, such as glucocorticoids, may have varied effects on the GI tract in patients with COVID-19 (4).

The high expression of ACE2 on the GI tract may explain the existence of GI symptoms in COVID-19 patients (5, 6). ACE2 is specifically expressed in enterocytes which are mainly from the gastric mucosa of COVID-19 patients previously infected with *Helicobacter pylori* (*H. pylori*), suggesting that *H. pylori* infection may result in increased risks of COVID-19 infection (Zhang et al.). It is noteworthy that gut barrier integrity was found to be positively modulated by ACE2 through downregulation of stress-responsive pathways, so decreased expression of ACE2 in older patients with COVID-19 can attenuate their gut barrier defense, which provides a new insight into the mechanism of SARS-CoV-2 invasion (Moon et al.). Given the potential multiple roles of ACE2 in the GI tract, researchers are not sure whether the declined ACE2 in the GI tract with age is related to SARS-CoV-2 infection. More clinical and basic studies are needed to explore the multiple roles of ACE2 in the GI tract.

Besides GI damages, liver injury has also been noted in COVID-19. The study by Lv et al. suggested that COVID-19 cases complicated with liver injury were more prone to becoming severe or critical, with a higher risk of death than those with normal liver function tests (LFTs). Jiang et al. also highlighted that SARS-CoV-2 infection may aggravate the hypercoagulability of pre-existing cirrhosis, which worsens the prognosis of COVID-19. In addition to D-dimer and total bilirubin (TBIL) (7), metabolic dysfunction-associated fatty liver disease (MAFLD) is also found as a risk factor of severe or critical COVID-19 (Hegyi et al.). Seow et al. also confirmed pre-existing liver diseases as a risk factor by analyzing the expression levels of ACE2 in five types of liver tissues *via* single-cell RNA-seq.

Inflammatory bowel disease (IBD), including ulcerative colitis (UC) and Crohn's disease (CD), is a chronic and relapse disorder in which immunosuppressive medications are frequently prescribed to induce and maintain remission. The COVID-19 pandemic has raised concerns about the management and therapy of IBD. On the one hand, as a series of biologic drugs widely used in IBD, anti-tumor necrosis factor α (anti-TNFα) agents are considered to increase the risk of virus infection including SARS-CoV-2 in IBD patients. However, Li et al. suggested that anti-TNFα treatment could potentially benefit IBD patients via downregulating the expression of colonic ACE2. On the other hand, COVID-19 has disrupted the management of IBD patients, posing a great challenge for gastroenterologists. A study by Qiu et al. demonstrated that the healthcare of IBD patients in epicentral areas is obviously impacted by COVID-19, including delayed lab tests/endoscopy procedures, delayed drug withdrawal, delayed biologics infusions, and postponed elective surgery. One way to counteract such a challenge is telemedicine (Qiu et al.), which in combination with virtual care, should be a promising future medical care paradigm in emergencies.

As a common examination in the GI department, GI endoscopy is a high-risk operation due to the potentially fecal-oral transmission of COVID-19, especially with its much higher transmissibility than influenza (8). Therefore, some precautions must be taken to contain virus transmission in this operation (Tian et al.). In addition, the psychological impacts of COVID-19 on GI endoscopists should not be overlooked (9), though they have adequate knowledge and awareness of occupational protection (Perisetti, Goyal, Sharma). Enough attention should be paid to the fear and anxiety of patients and medical staff for their psychological wellbeing during the COVID-19 pandemic (10).

We expect all the inspiring papers in this Research Topic “COVID-19 and the Digestive System” will contribute to improved prevention, diagnosis, and treatment for COVID-19.

## Author Contributions

CM and HZ wrote the manuscript. WD and BS edited the manuscript. HZ did the final checks of the manuscript and submitted it. All authors contributed to the article and approved the submitted version.

## Funding

HZ is supported by Grants from the 1.3.5 Project for Disciplines of Excellence, West China Hospital, Sichuan University (Grant No. ZYJC18037).

## Conflict of Interest

The authors declare that the research was conducted in the absence of any commercial or financial relationships that could be construed as a potential conflict of interest.

## Publisher's Note

All claims expressed in this article are solely those of the authors and do not necessarily represent those of their affiliated organizations, or those of the publisher, the editors and the reviewers. Any product that may be evaluated in this article, or claim that may be made by its manufacturer, is not guaranteed or endorsed by the publisher.
